# 7α-Hydroxypregnenolone, a key neuronal modulator of locomotion, stimulates upstream migration by means of the dopaminergic system in salmon

**DOI:** 10.1038/srep12546

**Published:** 2015-07-29

**Authors:** Shogo Haraguchi, Yuzo Yamamoto, Yuko Suzuki, Joon Hyung Chang, Teppei Koyama, Miku Sato, Masatoshi Mita, Hiroshi Ueda, Kazuyoshi Tsutsui

**Affiliations:** 1 Department of Biology and Center for Medical Life Science, Waseda University, Tokyo, Japan; 2 Department of Biology, Tokyo Gakugei University, Tokyo, Japan; 3 Field Science Center for Northern Biosphere, Hokkaido University, Hokkaido, Japan; 4 Current address: Demonstration Laboratory, Marine Ecology Research Institute, Niigata, Japan

## Abstract

Salmon migrate upstream against an opposing current in their natal river. However, the molecular mechanisms that stimulate upstream migratory behavior are poorly understood. Here, we show that 7α-hydroxypregnenolone (7α-OH PREG), a newly identified neuronal modulator of locomotion, acts as a key factor for upstream migration in salmon. We first identified 7α-OH PREG and cytochrome P450 7α-hydroxylase (P450_7α_), a steroidogenic enzyme producing 7α-OH PREG, in the salmon brain and then found that 7α-OH PREG synthesis in the brain increases during upstream migration. Subsequently, we demonstrated that 7α-OH PREG increases upstream migratory behavior of salmon. We further found that 7α-OH PREG acts on dopamine neurons in the magnocellular preoptic nucleus during upstream migration. Thus, 7α-OH PREG stimulates upstream migratory behavior through the dopaminergic system in salmon. These findings provide new insights into the molecular mechanisms of fish upstream migration.

Salmon display dramatic and complex life cycles, which are characterized by different types of migration: downstream migration, feeding migration and homing migration^1^. Homing migration comprises two main phases: an ocean phase when salmon migrate from the oceans into shore areas near their home-river, and a stream phase when they locate their main river and home tributary^2^. Salmon migrate upstream against an opposing current in their natal river. This upstream migration of salmon is energetically demanding because individuals have to pass a variety of natural barriers including waterfalls and rapid flowing water in their natal streams^3^,^4^,^5^,^6^. Electromyogram (EMG) recordings can be used an indicator of the swimming activity of salmon^7^,^8^ and particularly high levels of EMG activity were recorded during ascent of a pool-and-overfall fish ladder, indicating that high locomotor activity is required during upstream migration^9^. To date, however, the molecular mechanisms that underlie the increase in locomotor activity during this migratory phase are poorly understood.

It is well established that steroids can be synthesized *de novo* in the central and peripheral nervous systems. Such steroids are called “neurosteroids” and *de novo* neurosteroidogenesis from cholesterol is a conserved property in the brain of vertebrates (for reviews, see refs. 10, 11, 12, 13, 14, 15, 16). We recently found that amphibians and birds actively produce 7α-hydroxypregnenolone (7α-OH PREG), a previously undescribed bioactive neurosteroid^17^,^18^,^19^,^20^,^21^,^22^ in the brain. This novel bioactive neurosteroid acts as a neuronal modulator to increase locomotor activity in these vertebrates^17^,^18^,^20^. We further demonstrated that male newt brain exhibits marked seasonal changes in the synthesis of 7α-OH PREG and the expression of cytochrome P450 7α-hydroxylase (P450_7α_; gene name *Cyp7b*), a steroidogenic enzyme that catalyzes the formation of 7α-OH PREG from pregnenolone (PREG), and the maximum level occurs in the spring breeding period when locomotor activity of males increases^17^,^19^. Based on these observations, we hypothesized that the brain of salmon actively produces 7α-OH PREG, a neuronal modulator to stimulate locomotion, just before and during upstream migration to reach their spawning grounds.

To test our hypothesis, we conducted a series of experiments using adult chum salmon, *Oncorhynchus keta*. First, we identified 7α-OH PREG and P450_7α_ that catalyzes the formation of 7α-OH PREG in the salmon brain. Subsequently, we detected marked changes in 7α-OH PREG synthesis and concentration, and *Cyp7b* mRNA expression in the salmon brain during homing migration that comprises upstream migration. To investigate whether 7α-OH PREG is involved in upstream migration in the salmon, aminoglutethimide (AG), an inhibitor of cytochrome P450 side-chain cleavage enzyme (P450scc; gene name *Cyp11a*) that catalyzes the formation of PREG, a precursor of 7α-OH PREG, was intracerebroventricularly (icv) injected into the brain of salmon just prior to upstream migration for behavioral testing. 7α-OH PREG was also injected icv into the brain of AG-treated salmon for behavioral testing. To clarify the molecular mechanisms of 7α-OH PREG action on upstream migration, we further investigated the effect of central administration of 7α-OH PREG on dopamine concentration in the salmon brain. Finally, to better understanding the mode of 7α-OH PREG action on upstream migration of salmon, we investigated the localization of P450_7α_ and tyrosine hydroxylase (TH), a marker for dopamine neurons, in the salmon brain. This study provides new insights into the molecular mechanisms that underlie upstream migratory behavior in salmon from a novel standpoint.

## Results

### Identification of 7α-OH PREG in the salmon brain

**Figure 1:**
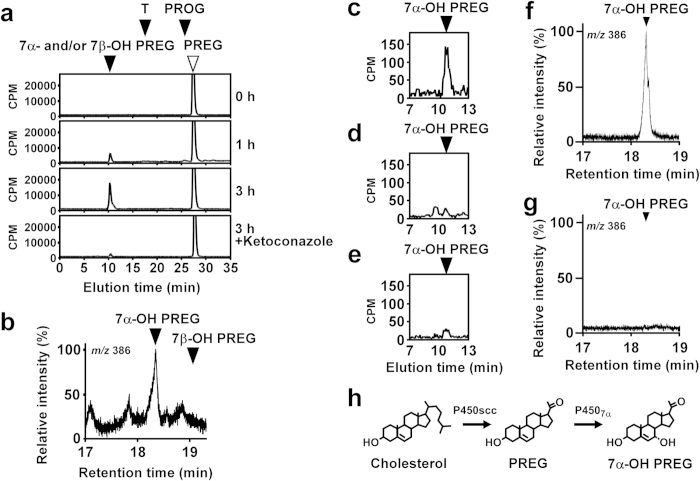
Identification of 7α-OH PREG and salmon P450_7α_ in the brain of chum salmon *Oncorhynchus keta*. (**a**) HPLC profile of an unknown metabolite of PREG by using a reversed-phase column. The brain homogenates were incubated with [^3^H]PREG, and the extracts were subjected to HPLC. The ordinate indicates the radioactivity measured in each HPLC fraction, and the arrowheads indicate elution positions of standard steroids, PREG, 7α-OH PREG, 7β-OH PREG, stereoisomer of 7α-OH PREG, PROG and T. (**b**) GC-selected ion monitoring (SIM) mass trace of *m/z* 386 in the extract from brain homogenates. The arrowheads indicate the retention times of 7α-OH PREG and 7β-OH PREG. The *m/z* 386 ion is a diagnostic ion of 7α- and 7β-OH PREG^17^,^18^,^19^,^20^,^21^,^22^. (**c**) 7α-OH PREG synthesis in COS-7 cells expressing the putative salmon *Cyp7b*. HPLC profile of [^3^H]7α-OH PREG extracted from COS-7 cells that were transfected with the putative salmon *Cyp7b* cDNA by pcDNA3.1/V5-His-TOPO and incubated with [^3^H]PREG. The ordinate indicates the radioactivity measured in each HPLC fraction, and the arrowhead indicates the elution position of standard 7α-OH PREG. (**d**) HPLC profile of an extract from COS-7 cells that were transfected with the putative salmon *Cyp7b* cDNA and incubated with [^3^H]PREG in the presence of 10^−4^ M ketoconazole, an inhibitor of cytochrome P450s. (**e**) HPLC profile of an extract from COS-7 cells that were transfected with pcDNA3.1/V5-His-TOPO expression construct alone and incubated with [^3^H]PREG. (**f**) GC-MS analysis of 7α-OH PREG. GC-MS SIM trace (*m*/*z* 386) of the extract from COS-7 cells that were transfected with the putative salmon *Cyp7b* cDNA and incubated with PREG. The arrowhead shows the peak corresponding to 7α-OH PREG. The *m*/*z* 386 ion is a diagnostic ion of 7α-OH PREG^17^,^18^,^19^,^20^,^21^,^22^. (**g**) GC-MS SIM trace (*m*/*z* 386) of the extract from nontransfected COS-7 cells. (**h**) The salmon brain expresses not only P450scc^29^ but also P450_7_α (present study), and produces 7α-OH PREG from cholesterol *via* PREG (present study).

First, we identified a previously undescribed fish neurosteroid from the brain of chum salmon by using biochemical techniques combined with high-performance liquid chromatography (HPLC) and gas chromatography/mass spectrometry (GC-MS) analyses. The initial finding was that an unknown fish neurosteroid was metabolized from PREG in the salmon brain. Salmon brain homogenates were incubated with tritiated PREG as a precursor, and radioactive metabolites were analyzed by reversed-phase HPLC. A major radioactive peak of the metabolite was detected approximately 18 min before the elution of PREG (Fig. 1a). In addition, some small radioactive peaks corresponding to progesterone (PROG) and testosterone (T) were also detected (Fig. 1a). Several nonradioactive steroids were used as reference standards for HPLC analysis, and 7α-OH PREG and its stereoisomer 7β-OH PREG exhibited the same retention time as the radioactive peak under a similar chromatographic condition (Fig. 1a). The radioactive metabolite corresponding to 7α- and 7β-OH PREG increased in a time-dependent manner (Fig. 1a), and the inhibitor of P450s, ketoconazole (10^−4^ M), reduced the metabolite (Fig. 1a). The metabolite of PREG was analyzed further by GC-MS. Trimethylsilyl ether derivatives of the authentic 7α- and 7β-OH PREG and the metabolite obtained from nonradioactive PREG were prepared and used in the GC-MS analysis. Based on GC-selected ion monitoring (SIM) analysis [mass/charge (*m*/*z*) 386], the metabolite had a retention time which was identical to 7α-OH PREG (18.3 min; Fig. 1b). Thus, the unknown fish neurosteroid converted from PREG in the salmon brain was identified as 7α-OH PREG (Fig. 1h).

### Identification of salmon P450_7α_, a steroidogenic enzyme producing 7α-OH PREG, and demonstration of its enzymatic activity in the salmon brain

To determine the mode of 7α-OH PREG synthesis, we identified a cDNA from brain tissue encoding a putative chum salmon P450_7α_ (gene name *Cyp7b*), a steroidogenic enzyme that produces 7α-OH PREG from PREG. A combination of 3′- and 5′-RACE produced a sequence, which we then compared with those of newt, quail, mouse and human *Cyp7b*^18^,^19^,^23^,^24^. The identified putative salmon *Cyp7b* cDNA had a full length of 2,908 bp (Supplementary Fig. S1). The putative salmon *Cyp7b* open reading frame started with a methionine at nucleotide 74 and terminated with a TGA codon at nucleotide 1,565, which encoded a protein of 497 amino acids (Supplementary Fig. S1). The deduced amino acid sequence of the open reading frame (497 amino acids) shared 52%, 53%, 43% and 47% identities with newt, quail, mouse and human *Cyp7b*, respectively. The putative salmon *Cyp7b* contained a highly conserved motif, FXXGXXXCXG(XXXA) of *Cyps*^25^ (see double underlining in Supplementary Fig. S1), which is thought to represent the heme-binding site with the arrangement of amino acids around the cysteine residue postulated to preserve the three-dimensional structure of this region for ligand binding^26^. Another domain (see underlining in Supplementary Fig. S1), which may be conserved in *Cyps* and is responsible for steroid interconversions^27^,^28^, also featured in salmon *Cyp7b*.

After the identification of salmon *Cyp7b* cDNA, we demonstrated the enzymatic activity of this putative salmon P450_7α_. The homogenate of COS-7 cells transfected with the putative salmon *Cyp7b* cDNA converted PREG to 7α-OH PREG by HPLC analysis (Fig. 1c), whereas the inhibitor of P450s, ketoconazole (10^−4^ M), reduced this metabolic process (Fig. 1d). COS-7 cells that were not transfected with the putative salmon *Cyp7b* cDNA did not convert PREG to 7α-OH PREG (Fig. 1e). Subsequently, 7α-OH PREG synthesis was confirmed by GC-MS analysis. The homogenate of COS-7 cells transfected with the putative salmon *Cyp7b* cDNA produced a metabolite that had the same retention time as 7α-OH PREG characterized by GC-SIM trace (Fig. 1f). COS-7 cells without transfection of the putative salmon *Cyp7b* cDNA did not convert PREG to 7α-OH PREG (Fig. 1g). It has been reported that the salmon brain expresses P450scc that catalyzes the formation of PREG from cholesterol^29^. Thus, the salmon brain expresses not only P450scc but also P450_7α_, and produces 7α-OH PREG from cholesterol *via* PREG (Fig. 1h).

### Changes in 7α-OH PREG synthesis and concentration, and *Cyp7b* mRNA expression in the salmon brain during homing migration

**Figure 2:**
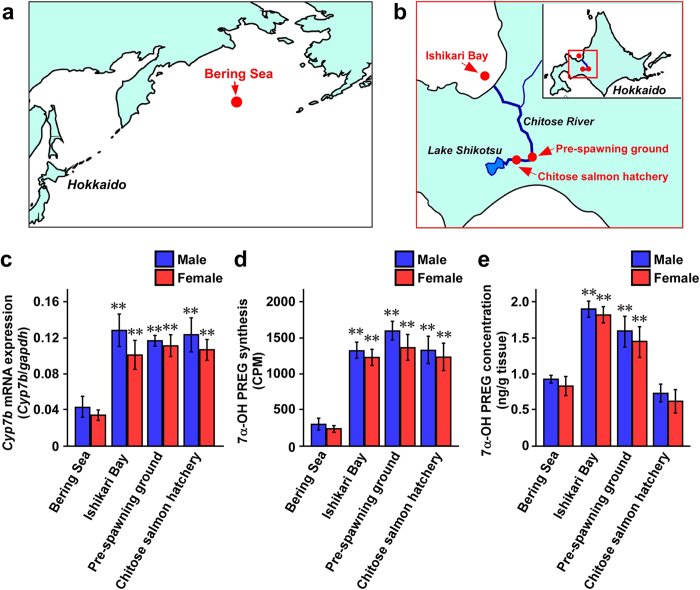
Changes in 7α-OH PREG synthesis and concentration, and *Cyp7b* mRNA expression in the brain of chum salmon *Oncorhynchus keta* during homing migration. (**a**,**b**) Salmon of both sexes were captured at the Bering Sea at the beginning of homing migration (**a**); Ishikari Bay, the entrance of upstream migration just prior to upstream migration (**b**); the pre-spawning ground during upstream migration (**b**); and the Chitose salmon hatchery, the goal of upstream migration just after upstream migration (**b**), Hokkaido, Japan. Canvas software (ACD Systems) was used to create the maps. (**c**) Changes in *Cyp7b* mRNA expression in the salmon brain during homing migration. (**d**) Changes in 7α-OH PREG synthesis in the salmon brain during homing migration. (**e**) Changes in 7α-OH PREG concentration in the salmon brain during homing migration. Each column and vertical line represent the mean ± s.e.m. of six independent samples. *P < 0.05 or **P < 0.01 versus Bering Sea.

To understand the physiological role of 7α-OH PREG during upstream migration, we examined changes in 7α-OH PREG synthesis and concentration, and *Cyp7b* mRNA expression in the salmon brain during homing migration that comprises upstream migration. Sexually mature adult chum salmon were collected from the Bering Sea to the Chitose salmon hatchery, Hokkaido, Japan during their homing migration (Fig. 2a,b). Fish of both sexes were captured from (i) the Bering Sea at the beginning of homing migration; (ii) the Ishikari Bay, the entrance of upstream migration just prior to upstream migration; (iii) the pre-spawning ground during upstream migration; and (iv) the Chitose salmon hatchery, the goal of upstream migration just after upstream migration (Fig. 2a,b). As shown in Fig. 2c, *Cyp7b* mRNA expression in the salmon brain of both sexes markedly changed at these different places during homing migration. *Cyp7b* mRNA was highly expressed in the brain of both sexes from the Ishikari Bay to the Chitose salmon hatchery compared with the Bering Sea (Fig. 2c). Similar changes in 7α-OH PREG synthesis in the brain of both sexes were detected (Fig. 2d). 7α-OH PREG concentration in the brain of both sexes also increased from the Ishikari Bay to the pre-spawning ground (Fig. 2e). However, 7α-OH PREG concentration in the brain was low at the Chitose salmon hatchery (Fig. 2e). These results indicate that 7α-OH PREG synthesis and concentration, and *Cyp7b* mRNA expression in the salmon brain increase just before and during upstream migration. In contrast to the brain, we did not detect a significant level of 7α-OH PREG in the plasma from the Bering Sea to the Chitose salmon hatchery during homing migration (Supplementary Fig. S2).

We further examined changes in the concentrations of PREG, a precursor of 7α-OH PREG (Supplementary Fig. S3a), and PROG, another metabolite of PREG (Supplementary Fig. S3a), in the brain of salmon during homing migration. PREG concentration in the brain did not change significantly from the Bering Sea to the pre-spawning ground and decreased at the Chitose salmon hatchery (Supplementary Fig. S3b). PROG concentration in the brain did not show any statistically significant change at different places during homing migration (Supplementary Fig. S3c).

### Reduction of upstream migration by inhibiting 7α-OH PREG synthesis and induction of upstream migration by 7α-OH PREG administration in salmon

**Figure 3:**
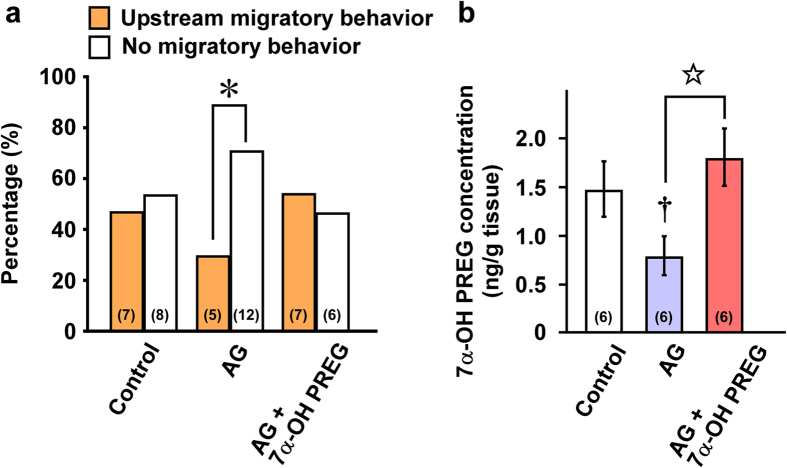
Effects of icv injection of AG or AG plus 7α-OH PREG on upstream migratory behavior of chum salmon *Oncorhynchus keta*, and 7α-OH PREG concentration in the salmon brain. Salmon of both sexes captured at the Ishikari Bay just prior to upstream migration were used and behavioral testing was conducted in an artificial river that consisted of upstream arm and pool. A gate prevented the test fish from entering the upstream arm. After the acclimation period, the gate was opened, and the water flow was introduced to the inlet of the arm for a 9-h period. Fish were allowed to move freely. The number of fish that stayed in the upstream arm (upstream migratory behavior) or the pool (no migratory behavior) was counted and expressed as a percentage of the total number of fish in each group. (**a**) Effects of icv injection of AG or AG plus 7α-OH PREG on upstream migratory behavior during the upstream migratory period. (**b**) Effects of icv injection of AG or AG plus 7α-OH PREG on 7α-OH PREG concentration in the brain. Control treatment consisted of an equal volume of vehicle (0.9% saline). Each column and vertical line represent the mean ± s.e.m. The number in parentheses indicates the number of fish. ^*^P < 0.05 upstream migratory behavior versus no migratory behavior in a. ^†^*P* < 0.05 versus control, ^☆^*P* < 0.05 AG versus AG plus 7α-OH PREG in b.

To investigate whether 7α-OH PREG is involved in the stimulation of upstream migratory behavior, 7α-OH PREG was manipulated in the brain of chum salmon captured at the Ishikari Bay just prior to upstream migration and subsequently behavioral analyses were conducted in an artificial river (see Methods). The number of fish that stayed in the upstream arm (upstream migratory behavior) or the pool (no migratory behavior) was counted and expressed as a percentage of the total number of fish in each group (Fig. 3a). As shown in Fig. 2, salmon at the Ishikari Bay showed high levels of 7α-OH PREG synthesis and concentration in the brain. To reduce the synthesis of 7α-OH PREG in the salmon brain, AG, an inhibitor of P450scc that catalyzes the formation of PREG, a precursor of 7α-PH PREG (Fig. 1h and Supplementary Fig. S3a), was injected icv into salmon. In the control group, the number of fish that exhibited upstream migratory behavior was almost half that of fish that were injected with icv vehicle (0.9% saline; Fig. 3a). However, icv injection of AG significantly (*P* < 0.05) decreased the number of fish that exhibited upstream migratory behavior and increased the number of fish that exhibited no migratory behavior (Fig. 3a). Thus, the number of fish exhibiting upstream migratory behavior was significantly (*P* < 0.05) lower than that of fish exhibiting no migratory behavior in the AG-treated group (Fig. 3a). By contrast, icv injection of 7α-OH PREG into AG-treated fish restored the number of fish that exhibited upstream migratory behavior (Fig. 3a). 7α-OH PREG concentration in the brain was decreased significantly (*P* < 0.05) by icv administration of AG, whereas this decrease in 7α-OH PREG concentration in the brain was restored significantly (*P* < 0.05) by icv co-administration of AG and 7α-OH PREG (Fig. 3b). These results indicate that 7α-OH PREG in the brain is important to stimulate upstream migratory behavior in this species.

### Comparisons of *Cyp7b* mRNA expression and 7α-OH PREG synthesis among different brain regions in salmon during upstream migration

**Figure 4:**
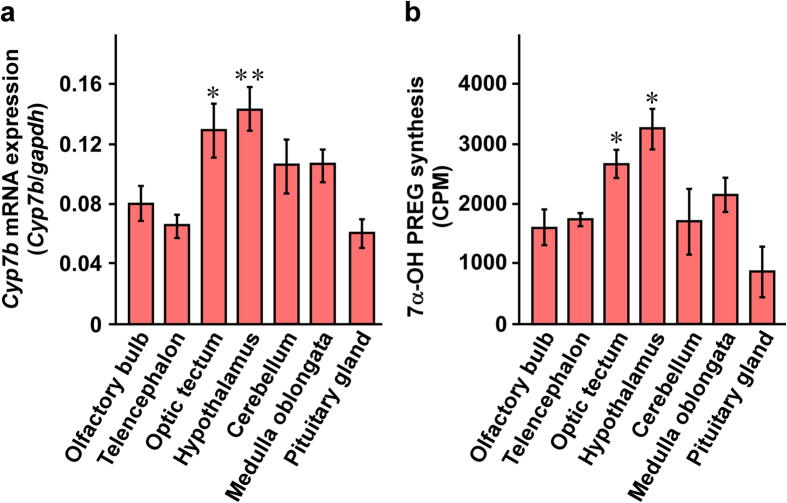
Comparisons of *Cyp7b* mRNA expression and 7α-OH PREG synthesis among different brain regions of chum salmon *Oncorhynchus keta*. Male salmon captured at the pre-spawning ground during upstream migration were used. Each column and vertical line represent the mean ± s.e.m. of six independent samples. *P < 0.05 or **P < 0.01 versus pituitary gland.

Data on the region-specific synthesis of 7α-OH PREG are required to understand the mode of action of this neurosteroid on upstream migratory behavior of salmon. Therefore, we compared the expression of *Cyp7b* mRNA and the synthesis of 7α-OH PREG among different brain regions of salmon captured at the pre-spawning ground during upstream migration. The salmon brain was subdivided into six regions: olfactory bulb, telencephalon, optic tectum, hypothalamus, cerebellum and medulla oblongata. As shown in Fig. 4a,b, *Cyp7b* mRNA expression and 7α-OH PREG synthesis were detected in all areas of the brain, but there were clear regional differences. The highest levels of *Cyp7b* mRNA expression and 7α-OH PREG synthesis were found in the optic tectum and hypothalamus compared to other brain regions, such as the olfactory bulb, telencephalon, cerebellum and medulla oblongata (Fig. 4a,b). *Cyp7b* mRNA expression and 7α-OH PREG synthesis in the pituitary gland were low in salmon during upstream migration (Fig. 4a,b).

### Enhancement of dopamine concentration by 7α-OH PREG administration in the salmon brain

**Figure 5:**
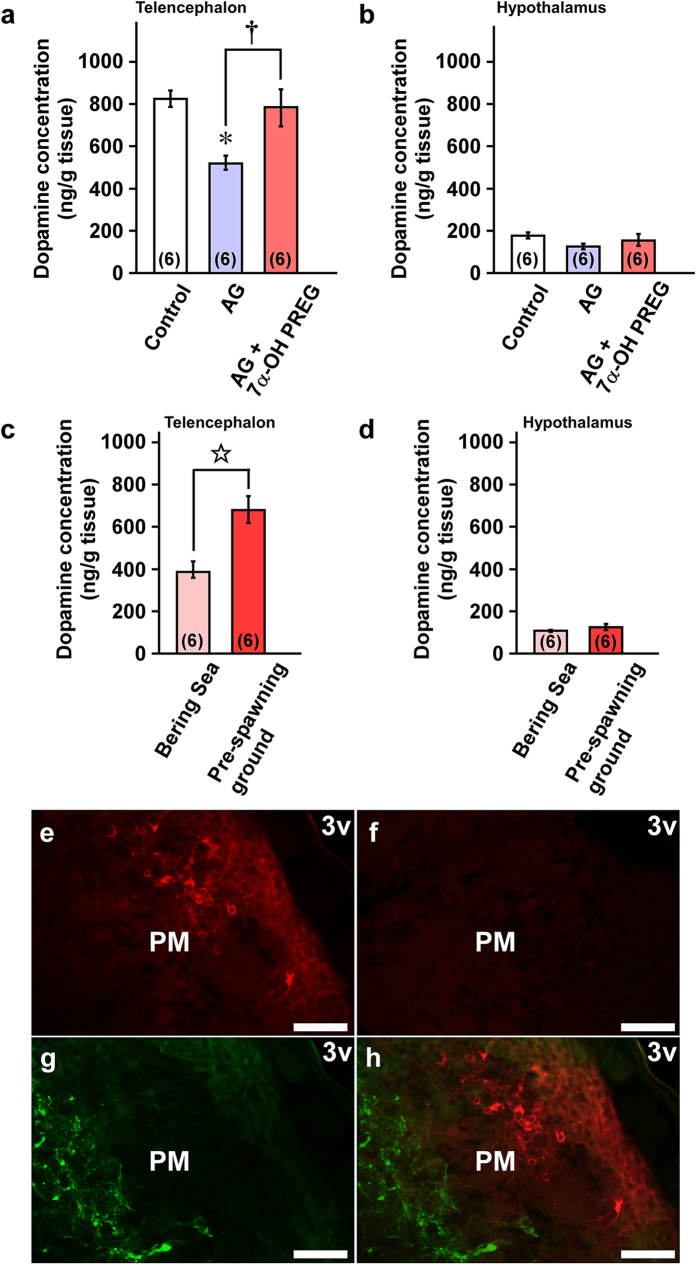
Enhancement of dopamine concentration by 7α-OH PREG administration and localization of P450_7α_ and TH in the brain of chum salmon *Oncorhynchus keta*. Effect of administration of AG or AG plus 7α-OH PREG on dopamine concentration in the telencephalon (**a**) and the hypothalamus (**b**) of salmon of both sexes captured at the Ishikari Bay just prior to upstream migration. Comparison of dopamine concentration in the telencephalon (**c**) and the hypothalamus (**d**) of salmon of both sexes captured at the Bering Sea and the pre-spawning ground. Each column and vertical line represent the mean ± s.e.m. The number in parentheses indicates the number of fish. *P < 0.05 versus control, ^†^*P* < 0.05 AG versus AG plus 7α-OH PREG in a. ^☆^*P* < 0.05 Bering Sea versus pre-spawning ground in c. (**e**–**h**) Localization of P450_7α_ and TH in the magnocellular preoptic nucleus (PM) in salmon captured at the pre-spawning ground. Immunostaining for P450_7α_ (**e**,**h**; *red*) and TH (**g**,**h**; *green*) in the PM. (**h**) Merged image between (**e**) and (**g**). (**f**) No immunostained cells were observed when the anti-salmon P450_7α_ antibody was preabsorbed with a saturating concentration of salmon P450_7α_ protein (10 μg/ml). Similar results were obtained in repeated experiments from four different salmon brains. Scale bars, 300 μm. PM, magnocellular preoptic nucleus; 3v, third ventricle.

We previously found that 7α-OH PREG increases the concentration of dopamine in the telencephalic region by acting on dopamine neurons localized in the diencephalic region in newts and quail^17^,^18^. Therefore, we next investigated whether dopamine concentration was altered by 7α-OH PREG in the brain of salmon captured at the Ishikari Bay just prior to upstream migration. AG-injected salmon had decreased telencephalic dopamine concentrations compared to the control group (*P* < 0.05; Fig. 5a). In addition, icv injection of 7α-OH PREG to AG-treated salmon increased dopamine concentration in the telencephalon compared with AG-treated salmon (*P* < 0.05; Fig. 5a). In contrast, 7α-OH PREG manipulation had no effect on hypothalamic dopamine concentrations (Fig. 5b). In addition, we compared the concentration of dopamine in the brain of salmon at the pre-spawning ground during upstream migration and the Bering Sea at the beginning homing migration. Telencephalic dopamine concentrations at the pre-spawning ground were much higher than that at the Bering Sea (Fig. 5c). In contrast to the telencephalon, hypothalamic dopamine concentrations did not show any statistically significant differences (Fig. 5d).

### Localization of P450_7α_ and TH in the salmon brain during upstream migration

Immuno-histochemical (IHC) analysis using anti-salmon P450_7α_ antibody was conducted to localize P450_7α_ in the brain of salmon at the pre-spawning ground during upstream migration. To confirm that this antibody recognizes the salmon P450_7α_ protein, we first performed Western blot analysis using the extract of COS-7 cells transfected with salmon *Cyp7b* cDNA. A single immunoreactive band (58 kDa) was detected (Supplementary Fig. S4). This band disappeared when the antibody was preabsorbed with salmon P450_7α_ protein (10 μg/ml; Supplementary Fig. S4).

In the olfactory bulb, immunoreactive cells were observed in the internal cell layer (ICL). In the telencephalon, immunoreactive cells were observed in the dorsal nucleus (Vd) and ventral nucleus (Vv) of the ventral telencephalic area. In the optic tectum, a large number of immunoreactive cells were observed in the stratum album central (SAC). An intense expression of P450_7α_ protein was detected in several hypothalamic regions. Clusters of the cells expressing P450_7α_ protein were localized in the following restricted regions in the hypothalamus: the magnocellular preoptic nucleus (PM; Fig. 5e), oculomotor nucleus (III), nucleus lateralis valvulae (NLV), and molecular layer (ML). In the cerebellum and medulla oblongata, some immunoreactive cells were detected in the medial longitudinal fascicle (mlf) and central gray (CG). No immunostained cell was observed when the anti-salmon P450_7α_ antibody was preabsorbed with salmon P450_7α_ protein (10 μg/ml; Fig. 5f).

To determine the mode of action of 7α-OH PREG on upstream migratory behavior, we further investigated the expression of tyrosine hydroxylase (TH), a marker for dopamine neurons, around the P450_7α_-expressing cells in the brain of salmon at the pre-spawning ground during upstream migration. Double immunolabeling showed that TH and P450_7α_ were expressed in two distinct cell populations that were close to each other in PM cells in the hypothalamus (Fig. 5h). TH immunoreactive fibers were also observed in the PM (Fig. 5g).

## Discussion

In this study, we first identified 7α-OH PREG in the salmon brain. Subsequently, we found that salmon brain expresses P450_7α_, a steroidogenic enzyme producing 7α-OH PREG from PREG. The expression of P450scc that produces PREG from cholesterol has been demonstrated in the salmon brain^29^. Taken together, it appears that the salmon brain produces 7α-OH PREG from cholesterol *via* PREG. Because salmon migrate upstream against an opposing current in their natal river^3^,^4^,^5^,^6^, high locomotor activity is required to complete upstream migration in salmon. Therefore, our aim in this study was to demonstrate that 7α-OH PREG, a neuronal modulator of locomotion, stimulates upstream migration in salmon.

We hypothesized that 7α-OH PREG is produced actively in the brain of salmon just before and during upstream migration. To test this hypothesis, we investigated changes in 7α-OH PREG synthesis and concentration, and *Cyp7b* mRNA expression in the salmon brain during homing migration comprising upstream migration. The synthesis and concentration of 7α-OH PREG in the brain were low in salmon at the Bering Sea at the beginning of homing migration and high in salmon at the Ishikari Bay just prior to upstream migration and the pre-spawning ground during upstream migration. In addition, similar changes in the expression of *Cyp7b* mRNA were evident in the salmon brain. In contrast, the concentrations of PREG, a precursor of 7α-OH PREG, and PROG, another metabolite of PREG, did not change in the brain of salmon from the Bering Sea to the pre-spawning ground. These results suggest that 7α-OH PREG plays an important role in the salmon brain to stimulate upstream migratory behavior during upstream migration.

*Cyp7b* mRNA expression and 7α-OH PREG synthesis were high in the brain from the Ishikari Bay to the Chitose salmon hatchery compared with the Bering Sea. 7α-OH PREG concentration in the brain also increased from the Ishikari Bay to the pre-spawning ground. However, 7α-OH PREG concentration in the brain decreased at the Chitose salmon hatchery. This discrepancy may be because the concentration of PREG, precursor of 7α-OH PREG, had decreased in the brain at the Chitose salmon hatchery (Supplementary Fig. S3b), resulting in low production of 7α-OH PREG, although *Cyp7b* mRNA expression and 7α-OH PREG synthetic activity were high in the brain at the Chitose salmon hatchery.

According to our previous study in newts, the synthesis and concentration of 7α-OH PREG changes during the annual breeding cycle, with a maximum level in the spring breeding period when locomotor activity increases^17^,^19^,^30^. In addition, we also reported that 7α-OH PREG is involved in enhancing the expression of sexual behavior of newts during the spring breeding period when locomotor activity increases^31^. To demonstrate the stimulatory action of 7α-OH PREG on upstream migratory behavior in salmon, 7α-OH PREG was manipulated in the brain of salmon captured at the Ishikari Bay just prior to upstream migration and behavioral analyses were conducted in an artificial river. We found that the inhibition of 7α-OH PREG synthesis in the brain by AG administration reduced the number of salmon that exhibited upstream migratory behavior. By contrast, 7α-OH PREG administration to AG-treated salmon rescued this behavior. These behavioral changes depended on changes in the 7α-OH PREG levels in the brain. Accordingly, 7α-OH PREG in the brain is possible to stimulate upstream migration of salmon to reach their spawning grounds. Because AG is an inhibitor of P450scc, administration of AG decreases not only 7α-OH PREG but also PREG and PROG. Our findings suggest that PREG and PROG in the salmon brain may not underlie upstream migration, as levels did not change in salmon from the Bering Sea to their pre-spawning ground. In addition to 7α-OH PREG, allopregnanolone (ALLO), a metabolite of PROG, is also known as a stimulator of locomotor activity in mammals^32^,^33^. ALLO is synthesized in the brain of teleost fish^34^,^35^. To reveal the detailed molecular mechanisms of upstream migration in salmon, we need to perform further experiments to clarify the action of ALLO on upstream migration.

To understand the mode of action of 7α-OH PREG during upstream migration, we first investigated the region-specific 7α-OH PREG synthesis and P450_7α_ expression in the salmon brain during upstream migration. The levels of 7α-OH PREG synthesis in the hypothalamus and optic tectum were higher than those in other brain regions, such as the olfactory bulb, telencephalon, cerebellum and medulla oblongata. P450_7α_-positive cells were localized mainly in the PM, III, NLV and ML in the hypothalamus and the SAC in the optic tectum. It is well known that neurosteroids are produced in neurons and glial cells in the brain in mammals, birds and amphibians^10^,^11^,^12^,^13^,^14^,^15^,^16^. In teleost fish, mRNAs of steroidogenic enzymes, P450scc, 3β-hydroxysteroid dehydrogenase/Δ^5^-Δ^4^-isomerase (3β-HSD; gene name *Hsd3b*), cytochrome P450 17α-hydroxylase/c17,20-lyase (P450_17α,lyase_; gene name *Cyp17*), cytochrome P450 aromatase A (P450aromA; gene name *Cyp19a*) and P450aromB (gene name *Cyp19b*), are mainly expressed in radial glial cells^34^,^35^. In addition, in fish it is thought that P450aromB-expressing radial glial cells are neural progenitor^36^,^37^,^38^. According to Diotel *et al*., radial glial cells are brain stem cells through the entire lifespan of fish, and it is suggested that some neurosteroids are implicated in this neurogenic process^34^,^35^. Additional studies are needed to elucidate the cell type of P450_7α_-expressing cells and the other function(s) of 7α-OH PREG in the brain of teleost fish.

Further investigation into the effect of 7α-OH PREG on dopamine neurons revealed that AG administration decreased dopamine concentration in the telencephalon, whereas 7α-OH PREG administration to AG-treated salmon rescued dopamine concentration in this brain region. In contrast, the hypothalamus showed no statistically significant change in dopamine concentration by 7α-OH PREG manipulation. Together these results indicate that 7α-OH PREG increases the release of dopamine in the telencephalon during upstream migration to induce upstream migratory behavior. In addition, P450_7α_ and TH were expressed in two distinct cell populations that were close to each other in PM cells in the hypothalamus, indicating that 7α-OH PREG may have a paracrine action on dopamine neurons in the PM. The coordinated action of several neurosteroidogenic enzymes is essential for understanding of neurosteroidogenesis. As for the production of 7α-OH PREG, the coordinated action of P450scc and P450_7α_ is required. Previous studies demonstrated that the brains of teleost fish express P450scc, and produces PREG, the precursor of 7α-OH PREG^34^,^34^,^35^. *In situ* hybridization showed that P450scc-positive cells were located in the PM^34^,^35^. Because the PM expressed P450_7α_, this nucleus may be the sites of 7α-OH PREG formation from cholesterol in the salmon brain.

It has been reported that dopaminergic neurons are localized in the hypothalamic region, and that they project into the telencephalic region in many vertebrates including fish and birds^39^,^40^,^41^. Here we have reported that 7α-OH PREG increases dopamine concentration in the telencephalic region^17^, which is known to be involved in the stimulation of locomotor behavior in vertebrates including fish^42^,^43^,^44^,^45^. Thus, 7α-OH PREG actively synthesized in the hypothalamus may act on dopamine neurons in the PM to induce dopamine release from their termini in the telencephalon and consequently stimulate upstream migratory behavior in salmon. Our previous study showed that the effect of 7α-OH PREG on locomotion is mediated by dopamine D_2_-like receptor in newts^17^. In addition, it has been reported that the role of the dopaminergic system in the stimulation of locomotor activity is mediated by dopamine D_2_-like receptor in the telencephalon in various vertebrates including fish^46^,^47^,^48^,^49^. These previous findings are in agreement with the present findings indicating that 7α-OH PREG stimulates upstream migratory behavior of salmon by means of the dopaminergic system.

## Methods

### Animals

Adult chum salmon *Oncorhynchus keta*, (3–5 years old; Supplementary Fig. S5) were collected during their homing migration from the Bering Sea to the Chitose salmon hatchery, Hokkaido, Japan. Fish of both sexes were captured by a longline in the Bering Sea (42°00’N-57°30′N, 179°00′E-180°00′E) [fork length (FL): 52.04 ± 6.5 cm; body weight (BW): 1.88 ± 0.66 kg; gonadosomatic index (GSI): gonad weight/body weight × 100): 0.28 ± 0.25 in male and 1.46 ± 1.23 in female] in June–July of 2008, by set nets at the Ishikari Bay (43°30′N, 141°35′E) [FL: 65.29 ± 3.67 cm; BW: 3.27 ± 0.65 kg; GSI: 3.92 ± 1.10 in male and 16.77 ± 3.24 in female] in September of 2008–2011, and by fence nets at the pre-spawning ground (42°83′N, 141°61′E) [FL: 64.56 ± 5.17 cm; BW: 2.79 ± 0.75 kg; GSI: 4.20 ± 1.41 in male and 16.92 ± 4.76 in female] and the Chitose salmon hatchery (42°83′N, 141°62′E) [FL: 63.08 ± 4.96 cm; BW: 2.68 ± 0.51 kg; GSI: spermiated in male and ovulated in female] in October of 2008–2011. After decapitation, brains and pituitary glands were flash frozen in liquid nitrogen and stored at –80 °C. Trunk blood was also collected into heparinized glass tubes and centrifuged at 1,800 × *g* for 20 min at 4 °C. Individual plasma was stored at −80 °C. The experimental protocol was approved in accordance with the Guide for the Care and Use of Laboratory Animals of Waseda University and Hokkaido University, Japan.

### Identification of 7α-OH PREG by biochemical analyses combined with HPLC and GC-MS

To identify a previously undescribed fish neurosteroid produced from PREG in the salmon brain, the radioactive metabolite of [^3^H]PREG (specific activity, 14 Ci/mmol (1 Ci = 37 GBq); PerkinElmer) was analyzed by HPLC using brain homogenates as described previously^17^,^18^,^19^,^22^. In brief, brain homogenates containing 200 mg of tissue from salmon were incubated in PBS containing 1 million cpm [^3^H]PREG, 0.24 mM NADPH, and 4% propylene glycol for 60 min at 25 °C. After incubation, steroids were extracted by ethyl acetate and subjected to HPLC analysis by using a reversed-phase column, LiChrospher 100 RP-18 (4.0 mm × 250 mm, Kanto). The column was eluted with a 30-min linear gradient of 40–70% acetonitrile at a flow rate of 0.7 ml/min, followed by an isocratic elution of 70% acetonitrile. The eluate was fractionated and counted in a flow scintillation analyzer (Radiomatic 525TR; PerkinElmer). To confirm the involvement of steroidogenic enzyme in the formation of the unknown fish neurosteroid, brain homogenates and [^3^H]PREG were incubated with ketoconazole (Sigma-Aldrich), an inhibitor of cytochrome P450s, at a final concentration of 10^−4^ M. The unknown fish neurosteroid was further examined in GC-SIM analysis as described previously^17^,^18^,^19^,^21^,^22^. In brief, 500 mg of salmon brain tissue was homogenized in 1 ml methanol/H_2_O (75:25; vol/vol) on ice. The homogenate was loaded on a C18 cartridge, and the steroid fraction was eluted with methanol and evaporated to dryness. Trimethylsilyl ether derivatives of the steroid fraction were prepared before GC-MS by reacting the dried sample with bis(trimethylsilyl)trifluoroacetamide (Wako Pure Chemical) at 60 °C for 30 min. For the identification of this unknown neurosteroid, a GC-MS system (GCMS-QP5000, Shimadzu) equipped with a CP-Sil 5CB capillary column (0.25 mm × 30 m, Varian) was used as previously described^17^,^18^,^19^,^21^,^22^. The column was maintained at 220 °C for 5 min, and then the temperature was raised to 300 °C at the rate of 5 °C/min. These biochemical analyses were repeated independently at least four times. Both 7α-OH PREG and its stereoisomer 7β-OH PREG, which were used as reference standards in these analyses, were purchased from Steraloids.

### Salmon *Cyp7b* cDNA cloning

Partial salmon *Cyp7b* cDNA was obtained from salmon brain cDNA by nested PCR using degenerate primers. The primers used for the PCR were as follows: sense primer 1, 5′-AGGAGASCTGGKGAGCCNCC-3′ (identical to nucleotides 148–168; GenBank accession no. AB824841), and antisense primer 1, 5′-CCAAYTGTTCTCTGGTGARG-3′ (complementary to nucleotides 1057–1076; GenBank accession no. AB824841). These PCR primers were designed on the basis of alignment of newt *Cyp7b* (AB374535), quail *Cyp7b* (AB329632), mouse *Cyp7b* (NM_007825) and human *Cyp7b* (NM_004820) mRNA sequences. The unknown sequences of 5′- and 3′-untranslated regions were analyzed by rapid amplification of cDNA ends methods as previously described^19^,^21^ with salmon *Cyp7b*-specific primers as follows: sense primer 2, 5′-TTCTGGGCMATGTATTATCTGC-3′ (identical to nucleotides 941–962; GenBank accession no. AB824841), sense primer 3, 5′-AGACCTCACCTTCACCAGAG-3′ (identical to nucleotides 1048–1067; GenBank accession no. AB824841), antisense primer 2, 5′-CTCCACTCGCTCTCCCCAGTAAGG-3′ (complementary to nucleotides 541–564; GenBank accession no. AB824841), and antisense primer 3, 5′-GATGACGTACGGATACAGCAACGG-3′ (complementary to nucleotides 314–337; GenBank accession no. AB824841).

### Enzymatic activity of salmon *Cyp7b* transfected in COS-7 cells

COS-7 cells were transfected with the putative salmon *Cyp7b* as described previously^18^,^19^ to assess its enzymatic activity. The full-length open reading frame of the putative salmon *Cyp7b* was amplified from salmon brain cDNA using the forward primer 5′-GCCGCCACCATGTTAGAGTTTGTTTTACC-3′ and the reverse primer 5′-TCAGGATCGACGTAGCCTGT-3′ and subcloned into the mammalian expression vector pcDNA3.1/V5-His-TOPO (Thermo Fisher Scientific). Positive colonies were selected and subcultured, and the plasmid DNAs were purified by the Wizard *plus* SV minipreps DNA purification system (Promega). COS-7 cells were supplied from Riken Cell Bank (Tsukuba, Japan) and maintained in DMEM (Sigma-Aldrich) supplemented with 10% (vol/vol) fetal bovine serum, penicillin (50 U/ml), streptomycin (50 μg/ml), and HEPES (10 mM, pH 7.4). Transfection was performed with the Lipofectamine 2000 (Thermo Fisher Scientific) as described previously^18^,^19^. After transfection, the cells were harvested, centrifuged (10,000 × *g* for 5 min at 4 °C), and stored at –80 °C. The cell homogenates were incubated with 1 million cpm of [^3^H]PREG for HPLC analysis or nonradioactive PREG for GC-MS analysis as described previously^18^,^19^. To confirm the enzymatic activity of the putative salmon P450_7α_, the cell homogenates and [^3^H]PREG were incubated with ketoconazole (Sigma-Aldrich), an inhibitor of P450s, at a final concentration of 10^−4^ M.

### Quantification of *Cyp7b* mRNA expression by real-time PCR

To measure the expression of *Cyp7b* mRNA in the salmon brain of both sexes during homing migration, real-time PCR was conducted by using the StepOnePlus system (Applied Biosystems) as described previously^22^. The PCR primers used for the amplification of salmon *Cyp7b* cDNA fragments were 5′-GCGGTGAATGAGATAAAGCAGTT-3′ (identical to nucleotides 1403–1425; GenBank accession no. AB824841) and 5′-CCTGTAGCGGATCTGGACATC-3′ (complementary to nucleotides 1532–1552; GenBank accession no. AB824841). The PCR primers for glyceraldehyde-3-phosphate dehydrogenase (GAPDH) were 5′-CAGCAATGCTTCATGCACAA-3′ (identical to nucleotides 508–527; GenBank accession no. NM_001123561) and 5′-CTCCACAGCTTTCCAGAAGGA-3′ (complementary to nucleotides 637–657; GenBank accession no. NM_001123561). *Gapdh* was used as the internal standard. The reaction mixture contained SYBR Green Real-Time PCR Mix (Toyobo), 400 nM each of forward and reverse primers, and 30 ng of cDNA in a final volume of 20 μl. PCR was run with a standard cycling program, 95 °C for 3 min, 40 cycles of 95 °C, 15 s; 60 °C, 15 s; and 72 °C, 15 s. An external standard curve was generated by a serial 10-fold dilution of cDNA obtained from the salmon brain, which had been purified, and its concentration was measured. To confirm the specificity of the amplification, the PCR products were subjected to a melting curve analysis and gel electrophoresis. The results were normalized to the expression of *gapdh* using the StepOnePlus 2.0 software (Applied Biosystems).

### Quantification of 7α-OH PREG synthesis by HPLC analysis

To measure the synthesis of 7α-OH PREG in the salmon brain of both sexes during homing migration, HPLC analysis was conducted as described previously^17^,^18^,^19^,^21^,^22^. Each homogenate containing 200 mg of the tissue was incubated separately with [^3^H]PREG for 60 min at 25 °C. After incubation, the extracted steroids were subjected to HPLC analysis.

### Quantification of 7α-OH PREG concentration by GC-SIM analysis

To measure the concentration of 7α-OH PREG in the salmon brain of both sexes during homing migration, GC-SIM analysis was conducted as described previously^17^,^18^,^19^,^21^,^22^. The extracted steroids derived from brain samples (500 mg tissue each) were applied to GC-SIM analysis. The concentration of 7α-OH PREG in the plasma of male salmon during homing migration was also measured by GC-SIM analysis^17^,^18^,^19^,^21^,^22^. Plasma samples (500 μl each) were used for GC-SIM analysis.

### Quantification of PREG and PROG concentrations by EIA

To measure the concentrations of PREG and PROG in the brain of male salmon during homing migration, the enzyme immunoassays (EIAs) were conducted as described previously^21^. In brief, 100 mg of salmon brain tissue was homogenized in 1 ml methanol/H_2_O (75:25; vol/vol) on ice. The homogenate was loaded on a C18 cartridge, and the steroid fraction was eluted with methanol and evaporated to dryness. The dried samples were dissolved in 500 μl EIA buffer containing 1% propylene glycol and vortexed for 10 min^21^. Each aqueous solution was divided into two aliquots for the measurements of PREG and PROG. PREG and PROG concentrations were assayed by using a pregnenolone EIA kit (Cayman Chemical) and a progesterone EIA kit (IBL), respectively.

### 7α-OH PREG manipulation and behavioral analyses

To investigate whether 7α-OH PREG is involved in the stimulation of upstream migratory behavior, 7α-OH PREG manipulation and behavioral analyses were conducted in September–November of 2010–2011 at the Toya lake station, Hokkaido University, Japan. Fish were collected just prior to upstream migration by set nets at the Ishikari Bay and subsequently transferred to the Toya lake station. A total of 45 fish of both sexes [FL: 65.00 ± 3.16 cm; BW: 3.01 ± 0.66 kg; GSI: 3.13 ± 0.67 in male and 18.12 ± 5.59 in female] were divided into 3 groups; vehicle (0.9% saline)-injected group (control group), AG-injected group (AG group) and AG plus 7α-OH PREG-injected group (AG plus 7α-OH PREG group). Each treatment solution (5 μl) was injected ICV into the brain of salmon through 0.3-mm diameter cannula that was implanted at the junction of the parietal and frontal bones in the cranial midline under anesthesia (0.005% clove oil; Wako Pure Chemical). AG (3 μg/5 μl) was injected 3 times every other day for 6 days (AG group), and then 7α-OH PREG (100 ng/ 5 μl) was injected one time (AG plus 7α-OH PREG group). Control salmon were injected with an equal volume of vehicle (0.9% saline).

Behavioral analyses were conducted in an artificial river that consisted of upstream arm (12 m × 0.6 m, 0.6 m water depth) and pool (3 m × 1.8 m, 0.6 m water depth) with an outlet at the end as described previously^50^,^51^. Before behavioral analyses, the test fish were kept for acclimation for 3 h in the pool. A gate prevented the test fish from entering the upstream arm. After the acclimation period, the gate was opened, and the water flow at 50 l/s was introduced to the inlet of the arm for a 9-h period. Behavioral testing was conducted between 7 pm and 4 am to avoid the influence of light intensity and the fish were allowed to move freely. The number of fish that stayed in the upstream arm (upstream migratory behavior) or the pool (no migratory behavior) was counted at the end of trial (4 am) and expressed as a percentage of the total number of fish in each group.

### Measurement of dopamine concentration by HPLC analysis

To investigate whether dopamine concentrations were altered by 7α-OH PREG in the salmon brain of both sexes, dopamine concentrations in the telencephalon and hypothalamus of salmon captured at the Ishikari Bay just prior to upstream migration were measured after behavioral analyses by HPLC-ECD (ECD-300, Eicom) as described previously^17^,^21^. Telencephalic and hypothalamic tissues (100 mg each) of salmon treated by vehicle alone, AG or AG plus 7α-OH PREG were homogenized in 0.2 M perchloric acid, maintained on ice for 30 min, and centrifuged at 15,000 × *g* for 15 min, and dopamine concentrations in the supernatant were measured. The concentrations of dopamine in the telencephalic and hypothalamic tissues were also measured at the Bering Sea at the beginning homing migration and the pre-spawning ground during upstream migration.

### Production of salmon P450_7α_ antibody

A synthetic oligopeptide corresponding to the 486–497 amino acid sequence of salmon *Cyp7b* (SDVQIRYRLRRS; GenBank accession no. AB824841) was produced as described previously^19^. For antibody production, keyhole limpet hemocyanin (Sigma-Aldrich) was coupled to the cysteine residues at the N-terminus of the synthetic oligopeptide by use of *m*-maleimidobenzoyl-*N*-hydroxysuccinimide ester (Sigma-Aldrich). Antibody against the oligopeptide of salmon P450_7α_ was generated in a rabbit by means of the lymph node injection technique^52^. For immunization, 100 μg hemocyanin-conjugated oligopeptide was dissolved in 100 μl saline, emulsified with an equal volume of Freund’s complete adjuvant (Dibco), and injected under Nembutal anesthesia. Starting two weeks after the first injection, four subcutaneous injections (100 μg each) were given in multi-sites on the dorsal surface of the rabbit every two weeks. Two weeks after the last injection, the rabbit was bled from the carotid artery and the serum was separated by centrifugation.

To confirm that this antibody recognized the appropriate antigen, Western blot analysis was performed on extract of COS-7 cells transfected with salmon *Cyp7b* cDNA as described above. The extract of COS-7 cells transfected with salmon *Cyp7b* cDNA were separated on a 12.5% SDS-polyacrylamide gel under reducing conditions and transferred to PVDF membranes (Hybond-P; GE Healthcare). The membrane was incubated with anti-salmon P450_7α_ antibody at 4 °C overnight and then for 1 h with goat-anti-rabbit IgG-horseradish-peroxidase conjugate diluted 1:1,000. An intense immunoreactive band was detected by using ECL prime Western blotting detection system (GE Healthcare). To control the specificity of the immunoreactive band, the primary antibody preabsorbed with the salmon P450_7α_ protein (10 μg/ml) was used.

### Immunohistochemical (IHC) staining with P450_7α_ antibody

IHC localization of P450_7α_ was performed as described previously^19^,^21^,^22^. In brief, chum salmon captured at their pre-spawning ground during upstream migration were terminated by decapitation. The brains were fixed in 4% (vol/vol) paraformaldehyde solution overnight, and they were soaked in a refrigerated 30% (vol/vol) sucrose solution in 0.1 M PB. Whole brains were frozen in OCT compound (Miles) and sectioned transversely at 20-μm thickness on a cryostat at –20 °C. After blocking nonspecific binding with 5% (vol/vol) normal goat serum and 1% BSA in PBS containing 0.5% Triton X-100, the sections were immersed overnight at 4 °C in 1:100 dilution of rabbit anti-salmon P450_7α_ antibody. The sections were then incubated for 60 min with Alexa Fluor 555 anti-rabbit IgG (Thermo Fisher Scientific) at a dilution of 1:1,000. After washing, the sections were mounted with mounting medium and visualized by using a fluorescence microscope (Leica).

### Double-labeling immunofluorescence for P450_7α_ and TH

Sections (20-μm thickness) were incubated at 4 °C overnight with rabbit anti-salmon P450_7α_ antibody and mouse anti-TH antibody (MAB318, Merck Millipore) in blocking solution. Then, the sections were incubated with Alexa Fluor 555 goat anti-rabbit IgG (1:1,000; Thermo Fisher Scientific) and Alexa Fluor 488 goat anti-mouse IgG (1:1,000; Thermo Fisher Scientific) secondary antibodies for 1 h at room temperature. Digitized pictures of the same microscopic field were captured for the two Alexa Fluors.

### Statistical analyses

Data were statistically analyzed with one-way ANOVA (when a normal distribution was found), and then Tukey-Kramer test was performed as a *post hoc* test. Student’s *t* test was also conducted to analyze for significance when the experiment consisted of only two groups. The behavioral testing was analyzed by using a chi-square test of independence. Significant difference was set at *P* < 0.05.

## Additional Information

**Acession codes:** The sequence reported in this paper has been deposited in the DNA Data Bank of Japan (DDBJ), European Molecular Biology Laboratory (EMBL), and GenBank database [accession no. AB824841 for cDNA sequence of Oncorhynchus keta cytochrome P450 7α-hydroxylase (gene name Cyp7b)].

**How to cite this article**: Haraguchi, S. *et al.* 7α-Hydroxypregnenolone, a key neuronal modulator of locomotion, stimulates upstream migration by means of the dopaminergic system in salmon. *Sci. Rep.* **5**, 12546; doi: 10.1038/srep12546 (2015).

## Supplementary Material

Supplementary Information
