# Supplementary material for: 7α-Hydroxypregnenolone, a key neuronal modulator of locomotion, stimulates upstream migration by means of the dopaminergic system in salmon
Source: Sci Rep. 2015 Jul 29;5:12546. doi: 10.1038/srep12546 (PMC4518220; doi:10.1038/srep12546)
Supplement: Supplementary Information [file srep12546-s1.pdf]

7α-Hydroxypregnenolone, a key neuronal modulator of locomotion, stimulates upstream migration by means of the dopaminergic system in salmon

Shogo Haraguchi[1,2], Yuzo Yamamoto[3,4], Yuko Suzuki[1], Joon Hyung Chang[1], Teppei Koyama[1], Miku Sato[1], Masatoshi Mita[2], Hiroshi Ueda[3], and Kazuyoshi Tsutsui[1,*]

[1]Department of Biology and Center for Medical Life Science, Waseda University, Tokyo, Japan
[2]Department of Biology, Tokyo Gakugei University, Tokyo, Japan
[3]Field Science Center for Northern Biosphere, Hokkaido University, Hokkaido, Japan
[4]Current address: Demonstration Laboratory, Marine Ecology Research Institute, Niigata, Japan.

***Corresponding author:** Kazuyoshi Tsutsui, Ph.D., Professor
Laboratory of Integrative Brain Sciences, Department of Biology, Waseda University
Center for Medical Life Science of Waseda University
2-2 Wakamatsu-cho, Shinjuku-ku, Tokyo 162-8480, Japan
Tel: 81 3 5369 7311; Fax: 81 3 3355 0316
E-mail: k-tsutsui@waseda.jp

```
GTTGATTTCAGCTGTCGATTACAACTTCCCTTGGCTTTATTTATTGTGGAGTGTCTGATC    60
ATTTCCAAACGAAATGTTAGAGTTTGTTTTACCATTGTTTCTCGGCTTTTTGGCTCTCTA   120
             M  L  E  F  V  L  P  L  F  L  G  F  L  A  L  Y     16
CTTACTCAGCGTGCGATTCAGGAGAACAAGGAGAGATGGGGAGCCTCCACTTATAAATGG   180
 L  L  S  V  R  F  R  R  T  R  R  D  G  E  P  P  L  I  N  G     36
TTGGATCCCGTTCGTTGGAAAGGCTTTGGAGTTTGGGAAGAATGCACATAGATTTCTGGC   240
 W  I  P  F  V  G  K  A  L  E  F  G  K  N  A  H  R  F  L  A     56
AGTGCATAAGGAGAAACATGGAGATGTATTTACTGTGCTGATTGCTGGCAAATACATGAC   300
 V  H  K  E  K  H  G  D  V  F  T  V  L  I  A  G  K  Y  M  T     76
CTTCATAATGAATCCGTTGCTGTATCCGTATGTCATCAAACATAAAAAACAGCTGGACTT   360
 F  I  M  N  P  L  L  Y  P  Y  V  I  K  H  K  K  Q  L  D  F     96
CCATGAGTTTTCTGACCAAGTGGCCCCCTTGACGTTCGGCTACCCCCCTGTCGGGAGCGG   420
 H  E  F  S  D  Q  V  A  P  L  T  F  G  Y  P  P  V  G  S  G    116
AAAGTTCCCCGGTATGAGCGAGCACATCCAGAGGTCCTTCCACCTTCTACAGGGCGACAA   480
 K  F  P  G  M  S  E  H  I  Q  R  S  F  H  L  L  Q  G  D  N    136
CCTCAATAACCTGACAGAGAGCTTGATGGGAAATCTCATGTTTGTGTTCCGACAAGACTA   540
 L  N  N  L  T  E  S  L  M  G  N  L  M  F  V  F  R  Q  D  Y    156
CCTTACTGGGGAGAGCGAGTGGAGGACTGAAAGTGTGTACCAACTCTGCAATTCGATCAT   600
 L  T  G  E  S  E  W  R  T  E  S  V  Y  Q  L  C  N  S  I  M    176
GTTTGAGGCTACTTTCCTGACCCTGTTTGGCAAGCCTGCCCATTCCAGCAGACACAGCGG   660
 F  E  A  T  F  L  T  L  F  G  K  P  A  H  S  S  R  H  S  G    196
AATGGTGACGCTTCGAGAGGACTTTGTCAAGTTCGACACCATGTTTCCCCTCCTCATCGC   720
 M  V  T  L  R  E  D  F  V  K  F  D  T  M  F  P  L  L  I  A    216
CAGGATCCCCATCTCTCTGCTGGGAGGAACCAAGGCCACTCGGGATAAACTGATCAACTA   780
 R  I  P  I  S  L  L  G  G  T  K  A  T  R  D  K  L  I  N  Y    236
CTTCCACCCTCAGAGAATGTCTGGATGGTCCAACACCTCAGGGTTCATAAAGGAAAGAGC   840
 F  H  P  Q  R  M  S  G  W  S  N  T  S  G  F  I  K  E  R  A    256
AGCAGTGCTTGAACAGTATGACTCCTTGGGAGATGTCGATAAAGCAGCTCATCATTTTGC   900
 A  V  L  E  Q  Y  D  S  L  G  D  V  D  K  A  A  H  H  F  A    276
CATTCTATGGGCGTCGGTGGGAAACACAGTCCCAGCCACCTTCTGGGCCATGTATTACCT   960
 I  L  W  A  S  V  G [N] T  V  P  A  T  F  W  A  M  Y  Y  L    296
GCTGACGCACCCAGAGGCCCTTGCAGTCGTGCGTGAGGAGATCCATGGTGTCCTGCTGGT  1020
 L  T  H  P  E  A  L  A  V  V  R  E  E  I  H  G  V  L  L  V    316
CTCAGGAATAGAAACACACCACAACAGAGACCTCACCTTCACCAGAGAACAGCTGGACAG  1080
 S  G  I  E  T  H  H  N  R  D  L  T  F  T  R  E  Q  L  D  S    336
CCTACTGAATCTGGAAAGCTCCATAAATGAAAGTCTGCGGCTGTCTTCAGCCTCCATGAA  1140
 L  L  N  L  E  S  S  I  N  E  S  L  R  L  S  S  A  S  M  N    356
CATCCGCATGGCCCAGGAGGACTTCAGCTTGCGGCTGGAGGGAGAGCGCTCCATTGGAGT  1200
 I  R  M  A  Q  E  D  F  S  L  R  L  E  G  E  R  S  I  G  V    376
GAGGAAAGGAGATCTCATCTCCCTGTATCCCCAGAGCATGCACATGGACCCCGGGATCTA  1260
 R  K  G  D  L  I  S  L  Y  P  Q  S  M  H  M  D  P  G  I  Y    396
CGAGAATCCAGAGAGCTACAAGTTTGACCGATACATCGAAAATGGAAAGGAAAAGACAGA  1320
 E  N  P  E  S  Y  K  F  D  R  Y  I  E  N  G  K  E  K  T  D    416
CTTCTATAAGGACGGCCAGAAGCTGAAGAACTACCGGATGTCTTTCGGCTCGGGCTCCAC  1380
 F  Y  K  D  G  Q  K  L  K  N  Y  R  M  S  F  G  S  G  S  T    436
TAAGTGCCCAGGGAGGTACTTTGCGGTGAATGAGATAAAGCAGTTCCTCTCTCTGCTGCT  1440
 K  C  P  G  R  Y  F  A  V  N  E  I  K  Q  F  L  S  L  L  L    456
ACTCTACTTTGACATGGATGTGTTGGAGGGGCAGAAGCCGTGTACCCTGGACCCCAGCCG  1500
 L  Y  F  D  M  D  V  L  E  G  Q  K  P  C  T  L  D  P  S  R    476
TGCTGGCCTGGGCATCCTGCTCCCTACCAGTGATGTCCAGATCCGCTACAGGCTACGTCG  1560
 A  G  L  G  I  L  L  P  T  S  D  V  Q  I  R  Y  R  L  R  R    496
ATCCTGAGAGGAGGAGGACATCTGTATATGAACAAACACTAGAGGGCTGAGCCCTGTGTG  1620
 S  *
GCTAATGGATTTGATTTCTCATCGGATGCATCCCAAATGGTACCCTATTTATTCCCTACA  1680
TAGTGCACTACTTTAGACCAGGAACCATAGGGCTTTTGTCAAAAGTACTGCACTATATTG  1740
GCAATGGTGTGCCATTTGGGACACACACATGGATTATTATTCACACAAGTAGCTATTGAC  1800
TGAACGGCAATGCATTGCTAATTTGTCAATGTATTATATTAGGACAGACTCTAACAATGG  1860
GCAGGATTGGCGAATCTGCTAGCTAGGGACCTTTGGTGTTTTATCCCATAGGACAGAATG  1920
ATCCATTAATTGTTAACAGTTTTTGCTGATTATAACAGATAGTATTTTATTTGAATACTA  1980
AAAATATGTTTGTTTATTATGCCTTGCATTATAAAACCATATATACCAGGGTATATAGGG  2040
GCCCTGGATTAATGTTGCAAAATGAAATAATATTTCATTTATATATATTTTTTGAAATAG  2100
GAACTGTCAGGGTCTCAACTTCCTGTTGAGTTAGAATATTAGAATACACAATGTGCAATT  2160
TCAAAATGTGTTTGTGCATCAGCAGTTCAGCTCTTATGTCAGTCACTCAATTACCTCATG  2220
TCAGCAATACATTTTTTTGATTGGTAAGTTAGTCTAGCCAGCTATCTTGACCTGTAGTAA  2280
TTGTGGTTGAATTACCGACCGGGACCCCCCATTGATTTTGTTAGTCACTCTCACTCAGAT  2340
ATCATATTAAAAAACGGCAAACATTTCTCTCCACCACATGACAAAATGTGTAGAATTGCA  2400
GGAAATGAACTCAAATTATTTTACATTTTTTCAATGTCAAAGTTGCCCATCCATGCTCTA  2460
TACTGTACAACAGTAGTTGAATCAAATAGTTGAACTATATTACAGGGTAAAAGGACAATG  2520
TGATAAAAGCTTTATGTTGTAAATATGGTGGTGTCCCTAAAACATGACTGATTTATTAGT  2580
TTCATTTTTCTATAACATTAACCAAATAACCAACCATTTAAGTTATACATTCAAATGTCT  2640
TTCCTATTTCAAGAGTCCTACAGAATATGTACAATTCATGAAATAAATGAAAACTGGACA  2700
AATCAGTTCTGCATGTGGCAGTAATTGTATTCCTTACATATTCCTTACATTTTTACCAAT  2760
AGTGACCTATTCTCTCAATTGGTCCAGTGAATCTGCAAGGTTCATACTCAGAGGAATTAT  2820
TTTGTTTGACCAACTCTCTTGTGTGAGTACAGCGTTCTGATCGTTGTTTGTCATTAATGC  2880
TGTCCCTGGTAATAAAATGATTCCATCC-polyA                            2940
```

Supplementary Figure S1 Nucleotide and deduced amino acid sequences of the putative chum salmon *Oncorhynchus keta Cyp7b* cDNA. The open reading frame is composed of 1,491 nt. The conserved Asn residue within the O_2-binding pocket of *Cyp7b* is indicated by a box[23]. Conserved residues of heme-binding domain (residues 431–444) are double underlined and postulated steroidogenic domain (residues 338–352) are underlined. The nucleotide sequence is deposited in the DDBJ, EMBL, and GenBank Sequence Databases under accession no. AB824841.

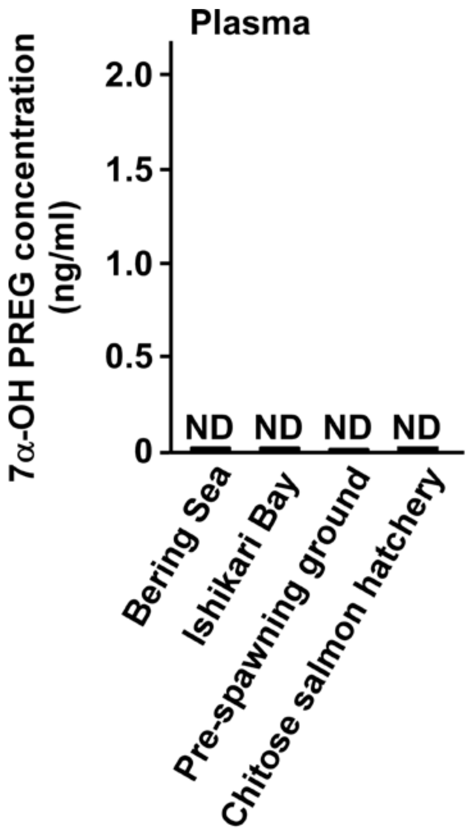

Supplementary Figure S2 Non detectable 7α-OH PREG concentration in the plasma of chum salmon *Oncorhynchus keta* during homing migration. Male salmon were captured at the Bering Sea at the beginning of homing migration; the Ishikari Bay, the entrance of upstream migration just prior to upstream migration; the pre-spawning ground during upstream migration; and the Chitose salmon hatchery, the goal of upstream migration just after upstream migration. Six independent samples at all places showed non detectable (ND) in plasma 7α-OH PREG.

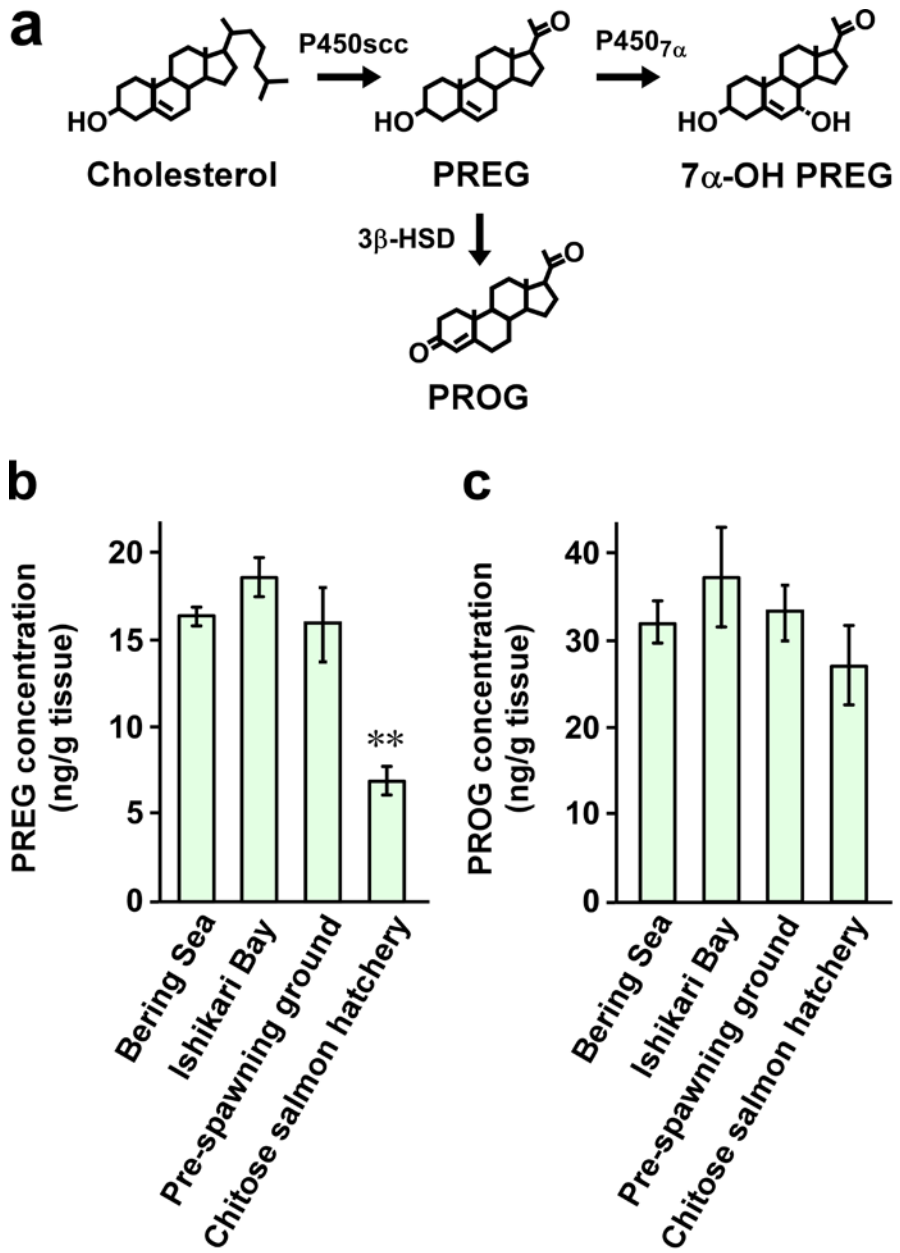

Supplementary Figure S3 Changes in PREG and PROG concentrations in the brain of chum salmon *Oncorhynchus keta* during homing migration. (a) Biosynthetic pathways of 7α-OH PREG and PROG from PREG. The salmon brain produces PREG, a precursor of 7α-OH PREG, 7α-OH PREG and PROG, another metabolite of PREG. (b, c) Changes in PREG and PROG concentrations in the salmon brain during homing migration. Male salmon were captured at the Bering Sea at the beginning of homing migration; the Ishikari Bay, the entrance of upstream migration just prior to upstream migration; the pre-spawning ground during upstream migration; and the Chitose salmon hatchery, the goal of upstream migration just after upstream migration. Each column and vertical line represents the mean ± s.e.m. of six independent samples. $^{**}P < 0.01$ versus Bering Sea.

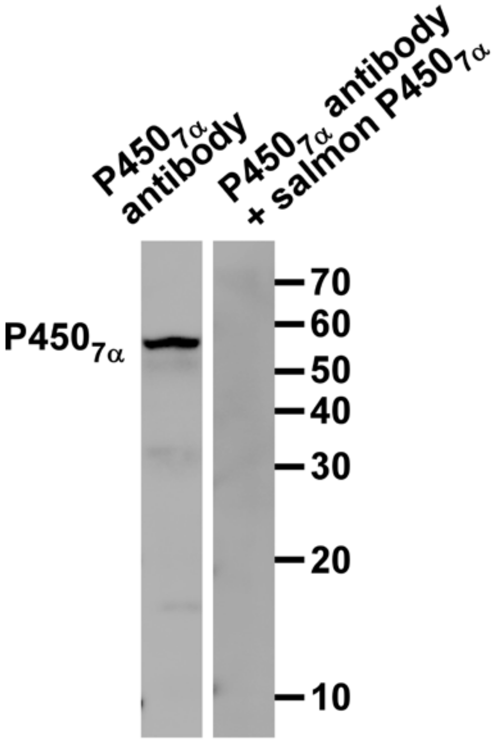

Supplementary Figure S4 Western blot analysis of the extract of COS-7 cells transfected with chum salmon *Oncorhynchus keta Cyp7b* cDNA with the anti-salmon $P450_{7\alpha}$ antibody.
Anti-salmon $P450_{7\alpha}$ antibody was preabsorbed with a saturating concentration of salmon $P450_{7\alpha}$ protein (10 μg/ml) for control. Similar results were obtained in repeated experiments using three different samples (data not shown).

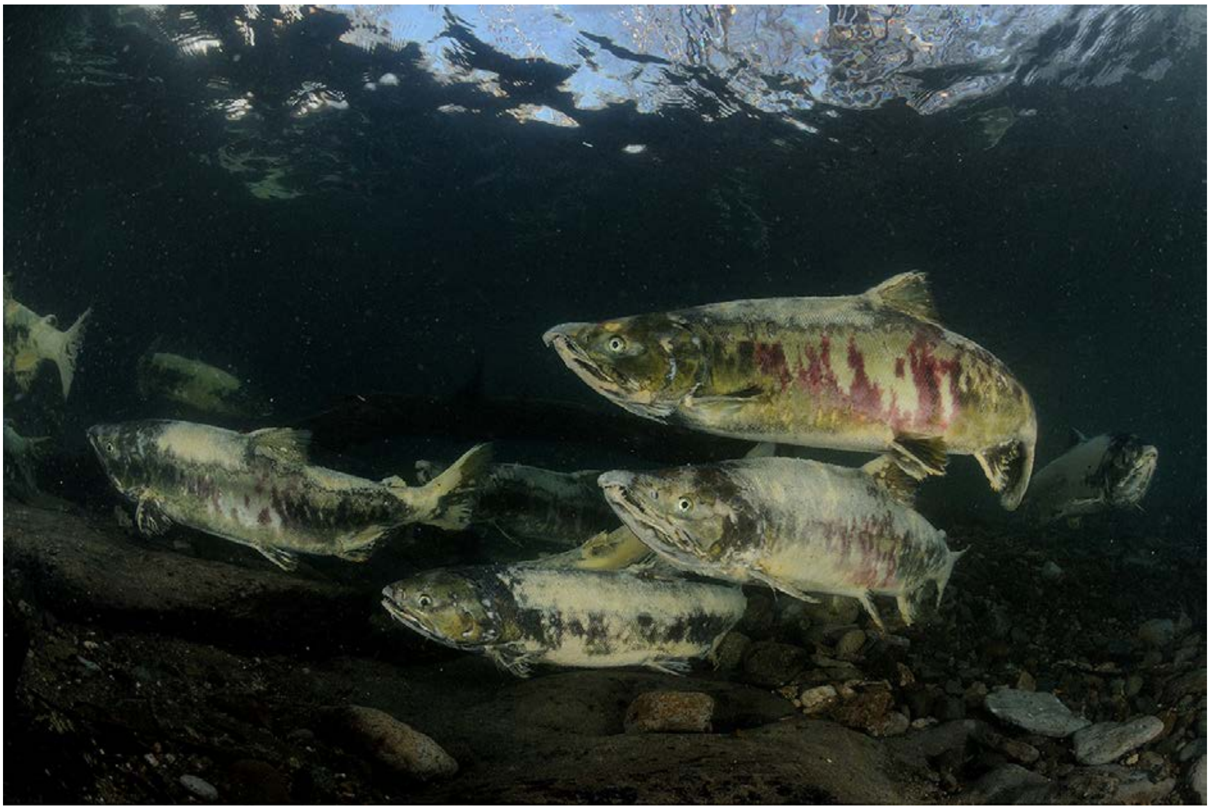

Supplementary Figure S5 Photograph of adult chum salmon (*Oncorhynchus keta*) approximately 3–5 years old that have just arrived at the Chitose salmon hatchery, Hokkaido, Japan from the Bering Sea. This picture was taken by Mr. K. Misawa.
